# Mechanisms and Pathways Linking Depression and Type 2 Diabetes Outcomes: A Scoping Review

**DOI:** 10.1155/jdr/5590413

**Published:** 2025-11-04

**Authors:** Andualem Derese, Sisay Sirgu, Yohannes Gebreegziabhere, Charlotte Hanlon

**Affiliations:** ^1^Department of Psychiatry, School of Medicine, College of Health Sciences, Addis Ababa University, Addis Ababa, Ethiopia; ^2^School of Public Health, College of Health and Medical Sciences, Haramaya University, Harar, Ethiopia; ^3^Department of Internal Medicine, St. Paul's Hospital Millennium Medical College, Addis Ababa, Ethiopia; ^4^Department of Nursing, College of Health Sciences, Debre Berhan University, Debre Berhan, Ethiopia; ^5^Division of Psychiatry, Centre for Clinical Brain Sciences, University of Edinburgh, Edinburgh, UK; ^6^Centre for Innovative Drug Development and Therapeutic Trials for Africa (CDT-Africa), College of Health Sciences, Addis Ababa University, Addis Ababa, Ethiopia

**Keywords:** depression, glycaemic control, psychosocial factors, self-efficacy, Type 2 diabetes mellitus

## Abstract

**Aims:**

People with diabetes experience a significantly higher prevalence of mental health issues, particularly depression. This adversely affects their diabetes management and overall health. This scoping review aims to develop a conceptual framework for understanding the connection between depression and diabetes outcomes globally, specifically focusing on intermediary factors that may influence this relationship.

**Methods:**

PubMed, EMBASE, PsycINFO, and Global Index Medicus were searched using relevant keywords on May 17, 2024. The inclusion criteria encompassed peer-reviewed studies involving adults diagnosed with Type 2 diabetes that assessed depression and analysed its impact on diabetes outcomes through various pathways.

**Results:**

The review identified 30 studies examining the association between depression and diabetes outcomes. Results indicate that while depression is linked to poorer diabetes outcomes like glycaemic control and complications of diabetes, the mechanisms are complex and often mediated by factors such as self-efficacy, social support, and diabetes-related distress. Notably, self-efficacy emerged as a critical mediator in the relationship between depression and self-management behaviours, which are known to be associated with diabetes outcomes. Furthermore, social support was identified as a protective factor that can reduce the adverse effects of depression on glycaemic control.

**Conclusions:**

Addressing mental health concerns in diabetes care is essential for improving patient outcomes. This review underscores the need for integrated interventions that consider psychosocial factors to enhance self-management and glycaemic control among individuals with Type 2 diabetes. Future research should focus on exploring these relationships in diverse populations to inform tailored strategies for effective diabetes management.

**Summary**

▪ Individuals with diabetes experience higher rates of mental health issues, particularly depression, which negatively impacts diabetes management and health outcomes.
▪ This scoping review identified 30 studies linking depression to poorer diabetes outcomes and developed a conceptual framework that highlights the complex mechanisms involved, including factors such as self-efficacy, self-management, illness perception, and social support.
▪ The findings emphasise the importance of addressing mental health in diabetes care.

## 1. Introduction

People living with diabetes mellitus (DM) have a considerably higher prevalence of mental health problems, especially depression, compared to those without the disease [1]. Factors contributing to this mental health burden include pain and disability arising from complications of the illness, concerns about how the illness will affect them, impacts of DM on relationships and work, and the challenges of living with a chronic condition [2]. To ensure a person's overall health and well-being, mental health concerns should be addressed. These include both diagnosed conditions or subclinical symptoms that may impact their ability to manage their condition effectively [3]. These psychosocial conditions include depression, anxiety, disordered eating, and cognitive impairment. Healthcare providers are advised to consider screening for these conditions as part of the initial assessment and at periodic intervals [3].

Depression is a mental health problem that is most often comorbid with diabetes [4–7]. In a systematic review of observational studies, 28% of people with Type 2 DM (T2DM) suffered from varying degrees of depressive disorders [8]. Another systematic review of 20 studies on the epidemiology of diabetes and depression showed a three times higher prevalence of depression among people living with Type 1 DM and about a two times higher prevalence among people living with T2DM compared to those without diabetes [5]. Similarly, another systematic review showed that depression and general distress comorbidity are higher among people living with DM compared to the general population and estimated the risk of developing depression in people with T2DM to be increased by 24% [6, 9].

Research has shown that comorbidity between diabetes and depression leads to increased morbidity, functional disability, complications, and mortality [4, 10–14]. Moreover, the occurrence of diabetes and depression comorbidity significantly increases the cost and burden of diabetes [4, 11]. A systematic review of the risk of mortality of people living with DM reported a 1.5 times higher risk of mortality related to depression [12]. Furthermore, the paper showed that depression was associated with an increased risk of mortality for all-cause and cardiovascular disease in people living with diabetes [12].

There is a paucity of information regarding the specific mechanisms by which depression negatively impacts the outcomes of individuals with T2DM. However, research suggests that depression can negatively impact self-efficacy, illness perceptions, and overall self-management behaviours [15], which in turn are associated with adverse DM outcomes. Understanding these mechanisms is important for designing effective interventions to address depression and ultimately reduce complications associated with DM.

This scoping review is aimed at developing a conceptual framework for understanding the relationship between depression and diabetes outcomes globally. The review considers intermediary factors that may influence the relationship between depression and diabetes outcomes.

## 2. Methods

### 2.1. Search Strategy

To identify relevant publications, PubMed, EMBASE, PsycINFO, and Global Index Medicus (GIM) databases were searched without restriction on publication date. Grey literature sources and trial registries were not searched, and the review was limited to peer-reviewed studies, as the primary aim of this scoping review was to synthesise the existing peer-reviewed evidence on mechanisms and pathways, rather than to identify all ongoing or unpublished studies.

The search was conducted on May 17, 2024. The search terms comprised key terms and MESH terms for depression, DM, self-efficacy, and self-management. The full search strategy for all databases is presented in File S1.

### 2.2. Eligibility Criteria

This review included peer-reviewed studies written in English that fulfilled the following criteria. Only studies published in English were included due to resource limitations for translation.

• Participants: adults (age ≥18 years) diagnosed with T2DM.
• Exposure: assessment of depression using either:
  o. Diagnostic categories of depressive disorders according to established criteria (e.g., DSM-5 and ICD-10), or
  o. Standardised depression scales measure depressive symptoms.
• Outcomes: Studies assessing the mechanisms or pathways through which depression influences diabetes outcomes. These outcomes include glycaemic control, self-management, development of diabetes complications, quality of life, mortality, healthcare utilisation and associated costs, psychosocial outcomes (e.g., social support, anxiety, and diabetes distress), and functional outcomes. For this review, ‘self-management' refers to the daily activities and behaviours undertaken by individuals to manage their diabetes. These include diet, physical activity, blood glucose monitoring, and medication adherence. ‘Self-care' is used synonymously with self-management. Self-management is treated as a mediator throughout. We distinguish papers that describe hypothesised mechanisms linking depression with self-management from those that have examined mechanisms linking depression and self-management, when studies did not also look at the association between self-management and diabetes outcomes.
• Study design: We included quantitative studies that explicitly analysed the pathways or mechanisms of depression affecting diabetes outcomes. This included analytic observational studies (e.g., cross-sectional, cohort, or case-control studies) or randomised controlled trials (RCTs) incorporating a mediation analysis or other methods to explore these pathways.
• Settings: Studies conducted in any global setting encompassing community and clinical settings.

### 2.3. Data Extraction

The primary author extracted the data using a checklist prepared in advance. Author, publication year, country, setting, study design, sample size, outcomes, measures, and key findings were extracted from each article.

### 2.4. Data Synthesis

Data synthesis was conducted to explore the mechanisms linking depression to key diabetes outcomes. The synthesis specifically focused on the pathways through which depression impacts diabetes outcomes. We extracted and synthesised results from studies employing path analysis and structural equation modelling (SEM) to analyse the relationships among the variables.

Synthesising the included studies' findings, we developed a preliminary conceptual framework that illustrates the complex interplay between depression, distress, and diabetes outcomes. This framework integrates key concepts and identifies potential causal relationships based on the evidence presented in the literature.

## 3. Results

**Figure 1:**
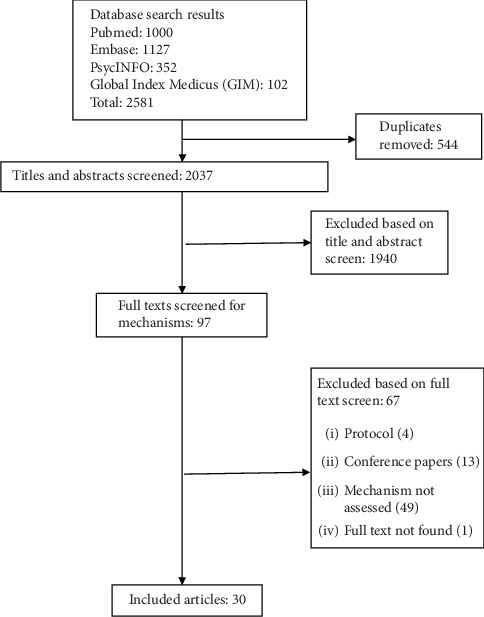
PRISMA flow diagram for pathways of depression and diabetes outcomes.

A total of 2581 articles were identified from the four databases. After removing 544 duplicates, 2037 titles and abstracts were screened, and 117 were selected for full-text review. Of these, 30 articles were eligible and included. Noneligible articles included brief reports, conference papers, and articles that did not assess mechanisms. The study selection process is illustrated in Figure 1.

Results are organised in two main parts, reflecting the types of study designs identified: (1) mechanisms linking depression and the diabetes outcome of glycaemic control and (2) mechanisms linking depression to self-management. Within each section, evidence for key psychosocial mediators (e.g., self-efficacy, social support, and adherence) is summarised. Detailed study characteristics are presented in Tables S1 and S2, which are organised thematically to align with these categories. Following each subsection, a summary table is provided (Tables 1 and 2, respectively) detailing the study characteristics, measures, and results. Studies are ordered by design strength (longitudinal > cross-sectional) within each table.

### 3.1. Results of Individual Studies

Most studies were from high-income countries (*n* = 21), with half (*n* = 15) from the United States. Nine articles reported on studies conducted in six LMIC countries. Except for four studies (one RCT and three longitudinal studies), most examined the interplay between depression, self-efficacy, and self-management through cross-sectional study designs. Most studies (*n* = 26) were conducted in clinical settings. The publication date ranged from 2004 to 2024. The sample sizes ranged from 99 to 917, and about two-thirds had a sample size of less than 300 participants. Most studies used SEM to assess the mechanisms.

#### 3.1.1. Glycaemic Control

Glycaemic control is crucial in managing diabetes and is often assessed using various biomarkers. One of the most common and reliable indicators is haemoglobin A1c (HbA1c) [46]. HbA1c is the form of haemoglobin that has bonded with glucose. This test retrospectively assesses average blood sugar control over 2–3 months [47]. The articles reviewed found that self-management [21, 22, 28], diabetes distress [25, 38], perceived control [24], self-efficacy [25], and fatalism [25] each had a direct effect on glycaemic control. Diabetes fatalism is a complex psychological cycle characterised by perceptions of despair, hopelessness, and powerlessness [48]. The relationship between depression and glycaemic control was inconsistent. Except for one study [20] demonstrating a direct link between depression and elevated HbA1c levels, the majority of the included studies did not find a significant direct association between depression and glycaemic control [16, 17, 19, 22, 26, 28]. However, many of these studies reported that depression had an indirect effect on glycaemic control. The mediators of this relationship included self-efficacy [17, 19, 21, 27], self-management [20–22, 28], social support [26], diabetes distress [16], and social comparison (or self-evaluations relative to others in the social environment, e.g., peers, family members, and media figures) [26].

A study by Cherrington et al. found a significant association between depressive symptoms and glycaemic control among men but not among women. This association among men was mediated by diabetes self-efficacy [27].

Diabetes-related distress had a direct effect [16, 25, 38] and an indirect effect on glycaemic control through diabetes self-efficacy, self-management, and perceived control [17, 21, 24]. Other socioeconomic factors, like income and higher social support, were also indirectly associated with glycaemic control. Social support had an indirect association with glycaemic control mediated through access to care and processes of care [25]. Three factors mediated the association between poverty and glycaemic control: cyclical representation of illness (perception of illness as unpredictable and cyclical), avoidance coping, and depressive symptoms [23]. One study found that avoidance coping was a full mediator, whereas depressive symptoms and a healthy diet partially mediated the association between education level and glycated haemoglobin (HbA1c) level [23]. The association between these socioeconomic factors and glycaemic control was no longer significant when each mediator was considered.

A study by Williams et al. evaluated the relationship between self-determination theory constructs (perceived competence and clinical autonomy support) to glycaemic control, depression, and patient satisfaction. The paper reported that autonomy support had an indirect effect on glycaemic control through perceived control [29].

Chiu and Du [18] investigated the influence of social support on the relationship between depression and glycaemic control in older Taiwanese adults. Their findings revealed a significant interaction effect. Among individuals reporting lower family and friend support, depressive symptoms at baseline (T1) positively predicted subsequent HbA1c levels, indicating a potential worsening of glycaemic control with increased depression. However, for participants with strong social support, while depressive symptoms (T1) and HbA1c were correlated at baseline, they did not predict each other 3 years later. This suggests that social support may buffer the negative consequences of depression on glycaemic control in older adults.

Overall, the evidence suggests that the association between depression and glycaemic control is predominantly indirect, mediated through psychosocial and behavioural factors such as diabetes distress, self-efficacy, self-management behaviours, and social support. The characteristics and findings of the included studies examining these pathways are summarised in Table 1. Detailed results, including specific measures and instruments used, are available in Table S1.

#### 3.1.2. Self-Management

Several psychosocial factors, including depression, diabetes distress, self-efficacy, diabetes knowledge, social support, and social–ecological support resources, were reported to have direct and indirect associations with either overall self-management score or some of the self-management components [17, 28, 31, 33, 38, 39, 41, 45, 49]. Depression, in particular, was directly linked to self-management behaviours, which are a key pathway through which depression influences diabetes outcomes. In addition, depression was indirectly related to self-management behaviours through self-efficacy, diabetes distress, social support by health workers, and social support by family and friends [19, 37, 38, 40, 41, 49]. However, there were also studies which found no significant association between depression and self-management behaviours [24, 30].

Other intraindividual variables, such as decreased emotional well-being, perceived personal control, and perceived situational control, were found to indirectly affect diabetes self-management activities through social–ecological support resources [43]. Similarly, Enggarwati et al. discovered a positive bidirectional association between social support and self-management activities. More significant social support led to increased engagement in self-management activities, and in turn, higher levels of self-management activities were associated with stronger social support [33]. Al-Amer et al. also showed that in Jordanian adults with Type 2 diabetes, social support and depression had a negative correlation, and social support was indirectly related to self-management behaviour through depression [36]. Diabetes fatalism had an indirect association with self-management behaviour through diabetes distress [38, 50].

Hudson et al. reported the effect of emotion and illness cognition on self-management behaviours. They found that participants who had concerns about their diabetes had more depression at 6 months of follow-up, indicating that cognitions may have a direct effect on emotional representations. Conversely, those participants who had higher baseline depression scores were more likely to believe that their diabetes was unpredictable at 6 months follow-up, which implies a direct effect from emotions to cognitions. Furthermore, baseline personal control beliefs directly affected adherence to diabetes self-management at 6 months follow-up [30].

A study by Gonzalez et al. examined the relationship between diabetes distress, medication adherence, self-efficacy, and perceived control in individuals with Type 2 diabetes. The results indicated that diabetes distress indirectly affected medication adherence, mediated by perceived control and self-efficacy, with only perceived control directly associated with better medication adherence [24]. Another study by Jiang et al. found that self-efficacy had a positive indirect effect on self-management behaviour, mediated by depression, with higher self-efficacy leading to lower depression and better self-management behaviour. The strength of this indirect effect might vary depending on age groups [31].

Another study found that self-efficacy mediated the relationship between depression and some components of self-management, like foot care among African Americans, but did not mediate among the Hispanic group [39]. Self-efficacy also mediates the relationship between depression and medication adherence [40, 42, 44]. Similarly, diabetes distress also has an indirect effect on medication adherence through perceived control and self-efficacy [24]. Osborn and Egede also found a direct inverse relationship between social support and depression and a direct positive relationship between social support and medication adherence [41]. Furthermore, depression also had an indirect relationship with medication adherence through perceived general barriers, perceived side effect barriers, and self-efficacy [44].

On the contrary, Gonzalez et al. reported that diabetes distress had no direct effect on self-management behaviours, suggesting that the impact of diabetes distress on self-management behaviours may be more complex and involve other mediating factors [24].

The findings indicate that self-management mediates the link between depression and glycaemic control. Depression is consistently associated with poorer self-management behaviours, both directly and indirectly through mediators including self-efficacy, diabetes distress, and social support. The key study characteristics and findings of included studies related to self-management outcomes are provided in Table 2. Detailed results are available in Table S2.

### 3.2. Conceptual Framework Synthesis

**Figure 2:**
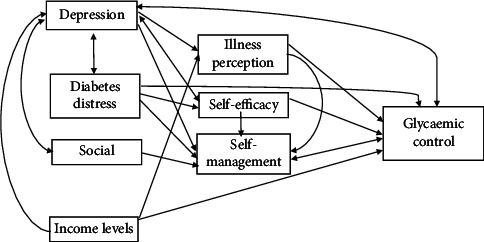
Conceptual framework of the pathways linking depression and Type 2 diabetes outcomes. Solid arrows represent unidirectional relationships supported by the evidence synthesised in this review. Double-headed arrows indicate bidirectional relationships. The framework highlights key mediators through which depression exerts its indirect effects on glycaemic control.

Synthesising the findings from the 30 included studies, we developed a conceptual framework (Figure 2) illustrating the mechanisms linking depression and diabetes outcomes. This framework integrates key mediators identified in this review.

## 4. Discussion

The results of this scoping review showed the complex relationship between depression and diabetes outcomes, mediated by various psychosocial and behavioural factors, including self-management, self-efficacy, illness perceptions, diabetes distress, socioeconomic status, and social support. The included studies used various study designs, including longitudinal and cross-sectional approaches across clinical and community settings.

Glycaemic control was influenced by depression and diabetes distress across several studies. Most studies included in this review found no significant direct association. But the effect was mediated through different factors. Diabetes distress was one of the mediators, where depressive symptoms exacerbate feelings of being overwhelmed by the demands of diabetes management, and this, in turn, directly impacts glycaemic control [16]. Similarly, a longitudinal study revealed that self-efficacy has an important role in mediating the relationship between diabetes distress and glycaemic control. This creates a cascading effect where higher diabetes distress influences depressive symptoms, which subsequently affect health behaviours like self-management and ultimately glycaemic control [17]. In contrast, another longitudinal study reported that while participants with higher depression scores were more likely to perceive their diabetes as unpredictable, this perception did not directly lead to changes in self-management behaviours or glycaemic control [30].

Existing evidence indicates that the relationship between depression and Type 2 diabetes outcomes appears to be bidirectional. Depression can negatively influence glycaemic control, medication adherence, and self-management behaviours [15, 45]. Evidence also suggests that poor glycaemic control, diabetes complications, and disease burden may contribute to the onset or worsening of depression [9, 30], suggesting a vicious cycle that complicates intervention efforts.

Self-management behaviours represented a key pathway linking depression and diabetes outcomes. Studies demonstrated that depression had a direct negative effect on self-efficacy, which in turn hindered healthier self-management practices [19, 37]. Some studies suggested that specific components of self-management, like medication adherence or foot care, might be differentially affected by depression [39, 41].

Similarly, social and contextual factors played a substantial role. A cross-sectional study emphasised the reciprocal relationship between social support and self-management activities, indicating that increased social support fosters engagement in self-management practices, which leads to better glycaemic control [33]. These findings indicated the importance of addressing psychological factors and enhancing social support to improve self-management and glycaemic control among individuals with Type 2 diabetes.

The studies reviewed demonstrate a clear relationship between depression and diabetes self-efficacy. For example, a cross-sectional study [19] found a significant direct effect of depression on self-efficacy. Good self-efficacy subsequently promoted healthier self-management practices, leading to favourable HbA1c levels. This is consistent with a study conducted in Malaysia, which indicated that both depression and diabetes distress negatively impacted self-efficacy [37]. Longitudinal studies reported that participants with higher baseline depression scores were more likely to perceive their diabetes as unpredictable over time [30]. This reciprocal relationship aligns with self-efficacy theory, suggesting that emotional states can enhance or undermine self-efficacy [32].

Despite these insights, several limitations must be acknowledged. Most included studies employed cross-sectional designs, limiting causal inferences regarding the relationships between variables. Another limitation is the variability in study design, measures, and definitions of outcomes and mediators, which complicates direct comparisons across studies. Additionally, most studies were conducted in high-income countries, suggesting a need for more research in LMICs to understand the unique challenges faced by these populations. Furthermore, the exclusion of non-English literature may have limited the diversity of included evidence, which should be considered when interpreting the proposed conceptual framework. Future research should prioritise longitudinal studies to establish causal relationships and explore community-based interventions that comprehensively address mental health and diabetes management. Additionally, it is important to consider the influence of contextual factors such as socioeconomic status, cultural beliefs, and healthcare access on the relationship between mental health and diabetes self-management. Qualitative studies can further illuminate the mechanisms underlying these relationships and identify culturally appropriate interventions.

Building upon the need to consider contextual factors highlighted in the limitations, this review also underscores how the broader sociocultural context significantly modifies the depression–diabetes relationship and its mediating pathways. Factors such as mental health stigma can hinder help-seeking and disclosure and create a barrier to effective depression management [34]. Culturally specific attitudes towards chronic illness and emotional distress (fundamentally shaping illness perceptions and coping strategies) [18], variations in the nature and availability of social support structures [33], and inequitable healthcare access across diverse populations [25] may fundamentally alter the pathways through which depression impacts self-management and glycaemic control identified in this review. The role of social mediators (e.g., comparison and perceived support) in these pathways is further evidenced in specific populations [26].

## 5. Conclusion

This scoping review underscores the complex pathways linking depression and Type 2 diabetes outcomes. Evidence identifies factors such as self-management, self-efficacy, illness perception, and diabetes distress as the main mediators. The relationship is often bidirectional, creating a challenging cycle for patients. Additionally, social support appears to moderate the effects of depression on diabetes outcomes by potentially buffering the negative impact of psychological factors on self-management and quality of life. Our conceptual framework integrates these findings. The evidence emphasise the necessity for integrated interventions that address both mental health issues and diabetes management comprehensively. Interventions aimed at enhancing self-efficacy, reducing diabetes distress, and fostering social support networks may be particularly beneficial in improving glycaemic control and overall health outcomes for individuals living with comorbid depression and diabetes.

## Figures and Tables

**Table 1:** Mechanisms linking depression and glycaemic control.

**First author, year**	**Country**	**Study design**	**Key findings on the pathway to glycaemic control**
Qian, 2023 [16]	United States	RCT	Depression → diabetes distress → higher HbA1c (Significant indirect effect)
Gao, 2022 [17]	China	Longitudinal	Diabetes distress & depression → self-efficacy → self-management → higher HbA1c (serial mediation)
Chiu, 2018 [18]	Taiwan	Longitudinal	Social support buffered the effect of depression on future HbA1c levels
Azami, 2019 [19]	Iran	Cross-sectional	Depression → lower self-efficacy → self-management → higher HbA1c (pathway statistically significant, but full indirect effect not)
Song, 2020 [20]	China	Cross-sectional	Depression → lower self-care → higher HbA1c (both direct and indirect effects significant)
Lin, 2017 [21]	China	Cross-sectional	Depression & diabetes distress → self-efficacy & self-management → higher HbA1c (indirect effects only)
Schmitt, 2016 [22]	Germany	Cross-sectional	Depression → suboptimal self-management → hyperglycaemia (full mediation)
Houle, 2017 [23]	Canada	Cross-sectional	Poverty → cyclical illness Rep./avoidance coping/depression → higher HbA1c (mediation)
Gonzalez, 2015 [24]	United States	Cross-sectional	Diabetes distress → perceived control → self-efficacy → higher HbA1c (indirect effect)
Walker, 2014 [25]	United States	Cross-sectional	Social support/stress/distress → self-care/access to care → higher HbA1c (indirect effects)
Arigo, 2014 [26]	Multicountry	Cross-sectional	Higher HbA1c → lower social support/social comparison → depression (mediation)
Cherrington, 2010 [27]	United States	Cross-sectional	Depression → lower self-efficacy → higher HbA1c (mediation found in men only)
Egede, 2010 [28]	United States	Cross-sectional	Depression → self-care → higher HbA1c (indirect effect only)
Williams, 2005 [29]	United States	Cross-sectional	Autonomy support → perceived competence → lower HbA1c (indirect effect)

**Table 2:** Mechanisms linking depression and self-management.

**First author, year**	**Country**	**Study design**	**Key finding on the pathway to self-management**
Hudson, 2016 [30]	UK	Longitudinal	Depression → unpredictable illness perception → (no direct effect on self-management). personal control beliefs directly improved self-management.
Jiang, 2023 [31]	China	Cross-sectional	Self-efficacy → lower depression → better self-management (mediation)
Bandura, 2022 [32]	UK	Cross-sectional	The negative impact of diabetes distress on mastery (self-management) was stronger at higher levels of depression (interaction effect).
Enggarwati, 2022 [33]	Indonesia	Cross-sectional	Reciprocal relationships: social support ⇄ depression ⇄ self-management ⇄ social support.
Egede, 2022 [34]	United States	Cross-sectional	Depression → negative illness perceptions (consequences subscale) → lower quality of life (mediation)
Derese, 2021 [35]	Ghana	Cross-sectional	Depression → lower social support (friends) → lower quality of life (mediation)
Al-Amer, 2018 [36]	Jordan	Cross-sectional	Social support → lower depression → better self-management Depression → self-efficacy → better self-management
Devarajooh, 2017 [37]	Malaysia	Cross-sectional	Depression & diabetes distress → lower self-efficacy → worse self-management (indirect effects)
Asuzu, 2017 [38]	United States	Cross-sectional	Depression & fatalism → higher diabetes distress → worse self-management (indirect effects) Higher diabetes distress → increased HbA1c (direct effect)
Hernandez, 2016 [39]	United States	Cross-sectional	Depression → self-management subcomponents of diet, physical activity, foot care, and smoking (direct effect) Depression → lower self-efficacy → worse foot care (mediation in African Americans, but not Hispanics)
Tovar, 2015 [40]	United States	Cross-sectional	Depression → lower self-efficacy → worse medication adherence (mediation). Social support by HCP also mediated; by spouse/significant other did not mediate this relationship.
Osborn, 2012 [41]	United States	Cross-sectional	Depression → lower social support → worse medication adherence (partial mediation)
Sacco, 2007 [42]	United States	Cross-sectional	Higher BMI → more diabetes symptoms/lower self-efficacy → depression (mediation). Self-efficacy mediated the adherence-depression link.
Harvey, 2006 [43]	Canada	Cross-sectional	Social-ecological support resources had a direct positive effect on self-management. Emotional well-being had an indirect effect via support resources.
Chao, 2005 [44]	United States	Cross-sectional	Depression → illness perceptions/lower self-efficacy → worse medication adherence (indirect effect)
McKellar, 2004 [45]	United States	Cross-sectional	Depression's impact on diabetes symptoms is primarily through its negative effect on self-management behaviours.

## Data Availability

The data utilised for this scoping review are the findings and information extracted from the published articles cited in the manuscript's reference list. No new primary data was generated. The information extracted is presented in the main text and summarised in the accompanying tables and supporting information.

## Nomenclature

ADDQoL: Audit of Diabetes Dependent Quality of Life
BIPQ: Brief Illness Perception Questionnaire
C-DES: Chinese version of the Diabetes Empowerment Scale
CES-D: Center for Epidemiological Studies-Depression Scale
CIRS: Chronic Illness Resources Survey
DASS-21: Depression Anxiety Stress Scale
DCP: Diabetes Care Profile
DDS: Diabetes Distress Scale
DES-SF: Diabetes Empowerment Scale-Short Form
DFS: Diabetes Fatalism Scale
DKQ: Diabetes Knowledge Questionnaire
DMSES: Diabetes Management Self-Efficacy Scale
DSMQ: Diabetes Self-Management Questionnaire
DUFSSQ: Duke-UNC Functional Social Support Questionnaire
DWBQ: Diabetes Well-Being Questionnaire
ESSI: Enrich Social Support Instrument
HbA1c: Haemoglobin A1c
INCOM: Iowa–Netherlands Comparison Orientation Measure
IPQ-R: The Revised Illness Perception Questionnaire
LDSES: Lorig's 8-Item Diabetes Self-Efficacy Scale
MADRS: Montgomery Asberg Depression Rating Scale
MDQ-SES: Multidimensional Diabetes Questionnaire Self-Efficacy subscale
MMAS: Morisky Medication Adherence Scale
MOS: Medical Outcomes Study Social Support Survey
MSPSS: Multidimensional Scale of Perceived Social Support
OPQoL: Older People's Quality of Life Questionnaire
PACIC: Patient Assessment of Chronic Illness Care
PAID: Problem Areas in Diabetes
PCDS: Perceived Competence for Diabetes Scale
PDSMS: Perceived Diabetes Self-Management Scale
PHQ: Patient Health Questionnaire
PHQ-9: Patient Health Questionnaire
PMS: Pearlin Mastery Scale
PSS: Perceived Stress Scale
REALM: Rapid Estimate of Adult Literacy in Medicine
RSC: Diabetes Fatalism Scale—Religious and Spiritual Coping
SDSCA: Summary of Diabetes Self-Care Activities
SEM: structural equation modelling
SPD: serious psychological distress
SSAS: Social Support Appraisal Scale

## Supporting Information

Supporting Information 1 File S1: Full search strategy for all databases.

Supporting Information 2 Table S1: Detailed characteristics, measures, and results of included studies examining pathways linking depression and glycaemic control.

Supporting Information 3 Table S2: Detailed characteristics, measures, and results of included studies examining pathways linking depression and self-management.
