# Supplementary material for: Mechanisms and Pathways Linking Depression and Type 2 Diabetes Outcomes: A Scoping Review
Source: J Diabetes Res. 2025 Nov 4;2025:5590413. doi: 10.1155/jdr/5590413 (PMC12614738; doi:10.1155/jdr/5590413)
Supplement: Supporting Information 1 — File S1: Full search strategy for all databases. [file 5590413.f1.docx]

Supplementary file 1: Search strategy for scoping review of mechanisms and pathways linking depression and type 2 diabetes outcomes

| Big terms |  | |
| --- | --- | --- |
| **Big term 1:**  Depression | Free terms | Depression [Title/Abstract] OR "major depressive disorder" [Title/Abstract] OR "Depression" [Mesh] OR "Depressive Disorder" [Mesh] OR "Depressive Disorder, Major" [Mesh] |
|  | MeSH terms | "Depression"[Mesh] OR "Depressive Disorder"[Mesh] OR "Depressive Disorder, Major"[Mesh] OR “Mental health” [Mesh] |
|  | Free term (Embase) | Depress* or dysthym* or mood disorder or mental health |
|  | Emtree terms | exp major depression/ or exp "mixed anxiety and depression"/ or exp depression/ or exp minor depression/ or exp bipolar depression/ |
|  | Psych-INFO terms | Depression or dysthymi$ or (depressi$ adj3 disorder$) or (depressi$ adj3 symptom$) |
| **Big term 2:**  Diabetes | Free terms | "diabetes mellitus, type 2" [Title/Abstract] OR "type 2 diabetes" OR "Diabetes Mellitus, Type 2" [Mesh] OR "Diabetes Mellitus" [Mesh] NOT "Diabetes Mellitus, Type 1" [Mesh] |
|  | MeSH terms | "Diabetes Mellitus"[Mesh] OR "Diabetes Complications"[Mesh] OR "Diabetes Mellitus, Type 2"[Mesh] |
|  | Free term (Embase) | non insulin dependent diabetes mellitus OR (Type* adj3 ("2" or "II" or two*) adj3 (diabete* or diabetic*)) OR (adult* onset* adj3 (diabete* or diabetic*)) OR ((Ketosis-resistant* or stable*) adj3 (diabete* or diabetic*)) OR ((Non-insulin* or Non insulin* or Noninsulin*) adj3 depend* adj3 (diabete* or diabetic*)) OR NIDDM.tw OR T2D |
|  | Emtree terms | exp diabetes mellitus/ |
|  | Psych-INFO terms | TYPE 2 DIABETES/ or (Type* adj3 ("2" or "II" or two*) adj3 (diabete* or diabetic*)) or (adult* onset* adj3 (diabete* or diabetic*)) or ((Ketosis-resistant* or stable*) adj3 (diabete* or diabetic*)) or ((Non-insulin* or Non insulin* or Noninsulin*) adj3 depend* adj3 (diabete* or diabetic*)) or NIDDM or t2d |
| **Big term 3:**  Self-care OR Self-efficacy OR Illness perception | Free terms | "self care" [Title/Abstract] OR "self management" [Title/Abstract] OR "Self-care behaviour" [Title/Abstract] OR "self-care" [Title/Abstract] OR "Self Care" [Mesh] OR "Self-Management" [Mesh] OR "illness cognition" [Title/Abstract] OR "illness cognition" [MeSH Terms] OR "illness perception" [Title/Abstract] OR "illness attribution" [Title/Abstract] OR "explanatory model" [Title/Abstract] OR "illness perception" [MeSH Terms] OR "Self Efficacy" [Title/Abstract] OR "Self-Efficacy" [Title/Abstract] OR "Self concept" [Title/Abstract] OR "Self Efficacy" [Mesh] |
|  | MeSH terms | "Self Care"[Mesh] OR "Self-Management"[Mesh] OR "Self Efficacy"[Mesh] |
|  | Free term (Embase) | self care or (self-care or self-management or self-monitor* or self-help) or self efficacy or illness perception |
|  | Emtree terms | exp self care/ or exp self concept/ or illness perceptions.mp. |
|  | Psych-INFO terms | self care or (self-care or self-management or self-monitor* or self-help) or self efficacy or illness perception or exp Self-Management/ or exp Self-Care Skills/ or exp Diabetes Mellitus/ or exp Health Behavior/ |
| **Final Search** | Big term 1 AND Big term 2 AND Big term 3 | |

***Abbreviation:*** MeSH, medical Subject Headings in MEDLINE
