# Supplementary material for: Mechanisms and Pathways Linking Depression and Type 2 Diabetes Outcomes: A Scoping Review
Source: J Diabetes Res. 2025 Nov 4;2025:5590413. doi: 10.1155/jdr/5590413 (PMC12614738; doi:10.1155/jdr/5590413)
Supplement: Supporting Information 2 — Table S1: Detailed characteristics, measures, and results of included studies examining pathways linking depression and glycaemic control. [file 5590413.f2.docx]

Supplementary Table 1. Detailed study characteristics and results for the mechanisms linking depression and glycemic control

| **Author; year; country** | **Study design; setting** | **Participants (Sample size and type)** | **Measures** | **Instruments** | **Results** |
| --- | --- | --- | --- | --- | --- |
| Qian Y; 2023; USA (29) | RCT; mixed (community and clinical) | 917; People with T2DM | Glycemic control | HbA1C | Using SEM:   - Depression at baseline leads to higher diabetes distress at the final assessment - Higher diabetes distress is then associated with poorer glycemic control (higher HbA1c) - There is a significant indirect effect of depression on HbA1c mediated by diabetes distress. |
|  |  |  | Depressive symptoms | PHQ-8 |  |
|  |  |  | Diabetes distress | Four items drawn from DDS |  |
| Gao Y; 2022; China (28) | Longitudinal study; clinical setting | 843; People with T2DM | Depressive symptoms | CES-D | - Self-efficacy played a key mediating role. Both diabetes distress and depressive symptoms indirectly affected dietary adherence, physical activity levels, and subsequent HbA1c levels through self-efficacy. - Depressive symptoms were a full mediator between diabetes distress and physical activity but not glycemic control - Diabetes distress has lessened the negative effect of depressive symptoms on diet, but not physical activity - Depressive symptoms solely mediated the negative effect of diabetes distress on diet - Significant serial mediation by depressive symptoms and self-efficacy in the relationship between diabetes distress and diabetes outcomes (diet, physical activity, and HbA1c). This suggests a cascading effect, where diabetes distress influences depressive symptoms, which in turn impacts self-efficacy, ultimately affecting health behaviours and glycemic control. |
|  |  |  | Diabetes distress | DDS |  |
|  |  |  | Self-efficacy | LDSES |  |
|  |  |  | Self-management | SDSCA |  |
|  |  |  | Glycemic control | HbA1C |  |
| Chiu CJ; 2018; Taiwan (33) | Longitudinal; community setting | 398; older adults (>51 years) with T2DM | Depressive symptoms | CES-D | Using cross-lagged SEM: Among individuals reporting lower family and friends support, there was a significant positive association between baseline depressive symptoms (T1) and subsequent HbA1C levels.  However, for participants with strong social support, depressive symptoms (T1) and HbA1C exhibited concurrent correlation at baseline but did not predict each other three years later. |
|  |  |  | Glycemic control | HbA1C |  |
|  |  |  | Family or friend support | Adapted from DCP |  |
| Azami G; 2019; Iran (27) | Cross-sectional; clinical setting | 142; People with T2DM | Glycemic control | HbA1C | Using SEM:  Depression had a significant direct effect on self-efficacy. Self-efficacy, in turn, promoted healthier self-management practices, which led to favorable HbA1c levels.  Statistically significant path coefficients from depression to self-efficacy, self-efficacy to self-management, and self-management to HbA1c. However, the indirect path from depression to HbA1c was not statistically significant. |
|  |  |  | Depression | CES-D |  |
|  |  |  | Self-efficacy | DMSES |  |
|  |  |  | Self-management | DSMQ |  |
| Song X; 2020; China (25) | Cross-sectional study; clinical setting | 428; People with T2DM | Anxiety | SAS | Using Hayes' PROCESS analysis*: 1. **Depression** is directly associated with **higher HbA1c 2. Depression** is also associated with **lower levels of self-care activities** (exercise and medication adherence) 3. **Lower self-care activities** are associated with **higher HbA1c 4.** There is a significant indirect effect of depression on HbA1c mediated by self-care activities |
|  |  |  | Depression | SDS |  |
|  |  |  | Self-care | SDSCA |  |
|  |  |  | Glycemic control | HbA1C |  |
| Lin K; 2017; China (18) | Cross-sectional, clinical setting | 254; People with T2DM (> 1 year after Diagnosis) | Depression | PHQ-9 | Using Generalized SEM:  1. Diabetes self-management had a **direct** effect on glycemic control  2. Depression had an **indirect** effect on glycemic control through diabetes self-efficacy and self-management  3. Diabetes distress had an **indirect** effect on glycemic control through diabetes self-efficacy and self-management  4. Site and duration of diabetes were significant covariates of glycemic control  5. Site and number of complications were significant covariates of diabetes self-management |
|  |  |  | Glycemic control | HbA1C |  |
|  |  |  | diabetes distress, | DDS |  |
|  |  |  | diabetes self-efficacy | C-DES |  |
|  |  |  | diabetes self-management | SDSCA |  |
| Schmitt A; 2016; Germany (19) | cross-sectional, clinical setting | 430; People with T2DM and T1DM | Depression | CES-D | Using SEM:  1. Suboptimal diabetes self-management significantly mediated the association between depressive symptoms and hyperglycaemia in people with diabetes (P < 0.001)  2. **NO** Significant direct association between depressive symptoms and hyperglycaemia |
|  |  |  | Diabetes self-management | DSMQ |  |
|  |  |  | Glycemic control | HbA1C |  |
| Houle J; 2017; Canada (31) | cross-sectional, clinical setting | 284; People with T2DM (> 3 months after Diagnosis) | Depression | PHQ-9 | Using the bootstrap method,  1. Cyclical representation of illness, avoidance coping, and depressive symptoms mediated the association between living in **poverty** and **HbA1c**  2. This association between living in poverty and HbA1c becomes non-significant when each mediator was considered, indicating complete mediation  3. Avoidance coping is a full mediator for the association between education level and HbA1c  4. Depressive symptoms and a healthy diet were partial mediators for the association between education level and HbA1c |
|  |  |  | Glycemic control | HbA1C |  |
|  |  |  | Self-management | SDSCA |  |
|  |  |  | Coping strategies | Brief- COPE |  |
|  |  |  | Quality of care | PACIC |  |
|  |  |  | Self-efficacy | DMSES |  |
|  |  |  | Illness representation | IPQ-R |  |
| Gonzalez JS; 2015; USA (23) | baseline data (cross-sectional) of an intervention study; Clinical setting | 142; People with T2DM | Diabetes-related Distress | DDS | Using Path analysis: (Medication adherence as an outcome)  1. Diabetes distress has an **indirect** effect on medication adherence through perceived control and self-efficacy  2. No significant direct effect of distress on adherence 3. Perceived control was independently and significantly associated with better medication adherence, and self-efficacy was not associated  4. Perceived control was independently associated with greater self-efficacy  (HbA1C as an outcome)  1. **Direct** effect of perceived control on HbA1C 2. Diabetes distress has an **indirect** effect on HbA1C through perceived control  3. There was no evidence of the direct effect of distress on HbA1C |
|  |  |  | Depression | MADRS |  |
|  |  |  | Self-efficacy; | B- IPQ |  |
|  |  |  | Glycemic control | HbA1C |  |
| Walker RJ; 2014; USA (22) | Cross-sectional, clinical setting | 615; People with T2DM | Fatalism | DFS | Using Path analysis:  1. **Direct** relationship between glycemic control and employment, fatalism, self-efficacy and diabetes distress. (lower HbA1c was associated with fewer hours worked, more fatalistic attitudes, more self-efficacy, and less distress diabetes were)  2. access to care and processes of care mediated the association between social and glycemic control  3. Diabetes distress, social support and perceived stress were associated with glycemic control mediated by **self-management**, **access to care** and **processes of care**). |
|  |  |  | Self-efficacy | PDSMS |  |
|  |  |  | Depression | PHQ-9 |  |
|  |  |  | Diabetes distress | DDS |  |
|  |  |  | Psychological Distress | SPD |  |
|  |  |  | Social support | MOS |  |
|  |  |  | Perceived stress | PSS |  |
|  |  |  | Medication Adherence | MMAS |  |
|  |  |  | Self-management | SDSCA; |  |
|  |  |  | Clinical | HbA1C |  |
| Arigo D; 2014; USA (26) (92%); from Canada, Europe, Southeast Asia, and the Middle East | cross-sectional, Online assessment | 185; People with T2DM | Depression | CES-D | Using Path analysis (the proposed path was from HbA1c to depression through social support and social comparison) 1. **Social support** significantly **mediated** the relationship between HbA1c and depressive symptoms (p=0.04)  2. **Social comparison** also significantly statistically **mediated** the relationship between HbA1c and depressive symptoms (p=0.03) 3. The relationship between social influences was unidirectional (from social comparison to social support; β=-0.12, p=0.02) 4. The direct path from HbA1c to depression **was no longer significant** when both mediators were included in the model (b=0.09, p=0.14) |
|  |  |  | Social comparison | IN-COM |  |
|  |  |  | Perceived Social support | SSAS |  |
|  |  |  | glycemic control, | HbA1c |  |
| Cherrington A; 2010; USA (30) | cross-sectional, clinical setting | 162; People with T2DM | Health literacy | REALM | Using Path Analysis:  1. A significant association between depressive symptoms and glycemic control was found for men (0.34, P < 0.01) but not for women (0.05, P = 0.59)  2. There is strong evidence that diabetes self-efficacy (as operationalized by the PDSMS) **mediates** the effect of depressive symptoms on glycemic control for **males** |
|  |  |  | Depression | CES-D |  |
|  |  |  | Perceived self-efficacy | PDSMS |  |
|  |  |  | Glycemic control | HbA1c |  |
| Egede L; 2010; USA (20) | cross-sectional, clinical setting | 126; People with T2DM | Depression | PHQ-9 | Using SEM,  1. Depression d**oes not have a direct effect** on glycemic control; instead, the relationship is **indirect via self-management** behaviours  2. More diabetes knowledge, social support, and less depressive symptoms were associated with performing diabetes self-management behaviour, explaining 24% of the variability in the diabetes self-management behaviour score  3. Diabetes self-management behaviour was marginally associated with glycemic control (r=−0.20, p=0.08 and r=−0.19)  # The Information— Motivation— Behavioural skills (IMB) model of health behaviour change was used |
|  |  |  | Diabetes knowledge | DKQ |  |
|  |  |  | Fatalistic attitudes | DFS-18 |  |
|  |  |  | Social support, | MOS |  |
|  |  |  | self-management behaviour | SDSCA |  |
|  |  |  | Glycemic control | HbA1C |  |
| Williams GC; 2005; USA (32) | cross-sectional, clinical setting | 634; People with T2DM | Perceived autonomy | HCCQ; | Using SEM,  1. Autonomy support had significant **direct** effects on perceived competence (β = 0.44, P< 0.01) and on patient satisfaction (β = 0.65, P< 0.01) **  2. Autonomy support had significant **indirect** effects (through perceived competence) on HbA1c (β =−0.10, P< 0.01) and depression (β =−0.18, P< 0.01)  3. Perceived competence significantly predicted both HbA1c (β =−0.22, P< 0.01) and depression (β =−0.22, P< 0.01), 4. Patient satisfaction significantly predicted depression (β =−0.13, P< 0.01). |
|  |  |  | Perceived competence | PCDS |  |
|  |  |  | Depression | PHQ-9 |  |
|  |  |  | Patient Satisfaction | 5-item scale from the ADA Provider Recognition program |  |
| **BIPQ** – Brief Illness Perception Questionnaire; **RSC** – Diabetes Fatalism Scale – Religious and Spiritual Coping; **SDSCA** – Summary of Diabetes Self‐Care Activities; **DMSES** – Diabetes Management Self‐Efficacy Scale; **DDS** – Diabetes Distress Scale; **C-DES** - Chinese version of the Diabetes Empowerment Scale; **DFS** - Diabetes Fatalism Scale; **SPD** - Serious Psychological Distress; **MOS** – Medical Outcomes Study - Social Support Survey; **PSS** – Perceived Stress Scale; **DKQ** – Diabetes Knowledge Questionnaire; **PCDS** – Perceived Competence for Diabetes Scale; **IN-COM** - Iowa-Netherlands Comparison Orientation Measure; **SSAS** – Social Support Appraisal Scale; **CES-D** - Center for Epidemiological Studies-Depression Scale; **REALM** – Rapid Estimate of Adult Literacy in Medicine; **PDSMS** – Perceived Diabetes Self-Management Scale; **MADRS** – Montgomery Asberg Depression Rating Scale; **PACIC** – Patient Assessment of Chronic Illness Care; **IPQ-R** -The revised Illness Perception Questionnaire; **DCP –** Diabetes Care Profile; **LDSES** - Lorig's 8-item Diabetes self-Efficacy Scale; | | | | | |

* Hayes' approach to mediation analyses uses bias-corrected bootstrap confidence intervals (bootstrapping = 10,000) to estimate and interpret the effect size of the direct and indirect effects of the independent variable on the dependent variable

** Used Self-determination theory (SDT)

*** The Baron and Kenny method is an analysis strategy for testing mediation analysis
