# Supplementary material for: Mechanisms and Pathways Linking Depression and Type 2 Diabetes Outcomes: A Scoping Review
Source: J Diabetes Res. 2025 Nov 4;2025:5590413. doi: 10.1155/jdr/5590413 (PMC12614738; doi:10.1155/jdr/5590413)
Supplement: Supporting Information 3 — Table S2: Detailed characteristics, measures, and results of included studies examining pathways linking depression and self-management. [file 5590413.f3.docx]

Supplementary Table 1. Detailed study characteristics and results for the mechanisms linking depression and self-management

| **Author; year; country** | **Study design; setting** | **Participants (Sample size and type)** | **Measures** | **Instruments** | **Results** |
| --- | --- | --- | --- | --- | --- |
| Hudson JL; 2016; UK (42) | Longitudinal observational study; clinical setting | 261; People with T2DM | Depression and anxiety symptoms | DWBQ | Using SEM   - Increased depression scores at baseline were associated with a belief that diabetes was unpredictable (timeline cyclical) at six months follow-up - Depression had no effect on diabetes self-management - Baseline illness perception about predictability of diabetes had no effect on diabetes self-management at six months - Personal control beliefs at baseline directly affected adherence to diabetes self-management at six months follow-up |
|  |  |  | Illness cognition | IPQ-R |  |
|  |  |  | Self-management | SDSCA |  |
| Jiang R; 2023; China (39) | Cross-sectional study; clinical setting | 240; People with T2DM | Self-management | SDSCA | Using Hayes' PROCESS analysis*:   1. Depression has a negative direct effect on self-management behaviour 2. Self-efficacy has a positive indirect effect on self-management behaviour mediated by depression. In other words, higher self-efficacy leads to lower depression, which in turn leads to better self-management behaviour 3. The strength of the indirect effect might vary based on age groups. |
|  |  |  | Self-efficacy | DMSES |  |
|  |  |  | Depression | PHQ-9 |  |
| McGuigan K; 2022; UK (48) | Cross-sectional study; clinical setting | 131; People with T2DM | Diabetes distress | PAID | Using Hayes' PROCESS analysis* There is an interaction effect between diabetes distress and depression. This means that the impact of diabetes distress on mastery (self-management) is stronger at higher levels of depression. |
|  |  |  | Mastery | PMS |  |
|  |  |  | Depression | HADS |  |
|  |  |  | Diabetes empowerment | DES-SF |  |
| Enggarwati P; 2022; Indonesia (38) | Cross-sectional; clinical setting | 94; People with T2DM | Depression | CES-D | Path analysis showed that: 1. Social support and depression symptoms exhibited a reciprocal relationship. Increased social support was associated with reduced depression symptoms, and conversely, fewer depressive symptoms were linked to stronger social support networks.  2. A positive bidirectional association emerged between social support and self-management activities. More significant social support fostered engagement in self-management, while increased self-management activities were linked to stronger social support.  3. Depression symptoms and self-management behaviours displayed a negative reciprocal effect. More depressive symptoms were associated with decreased self-management, and conversely, a decline in self-management activities was linked to increased depressive symptoms. |
|  |  |  | Self-management | SDSCA |  |
|  |  |  | Social support | DUFSSQ |  |
| Garcia A; 2022; USA (49) | Cross-sectional study; community setting | 75; People with T2DM | Depressive symptoms | CES-D | Using Hayes' PROCESS analysis*:  Depressive symptoms indirectly decreased quality of life (QoL) through the total score of the Illness Perception Scale. The study further examined the mediating role of the Consequences subscale, the only significant subscale predictor within the IPQ-R. The analysis revealed that depressive symptoms negatively impacted QoL through the Consequences subscale. |
|  |  |  | Illness perception | IPQ-R |  |
|  |  |  | Diabetes-Specific Quality of Life (QoL) | ADDQoL |  |
| Obo H; 2021; Ghana (50) | Cross-sectional study; clinical setting | 115; People with T2DM | Quality of life | OPQoL | Using Hayes' PROCESS analysis*   - Depression has a direct negative effect on quality of life - Depression can indirectly affect the quality of life through social support, particularly support from friends. i.e.: - Depression leads to decreased social support from friends. - Lower social support from friends is associated with poorer quality of life. |
|  |  |  | Social support | MSPSS |  |
|  |  |  | Depression and anxiety | DASS-21 |  |
| Al-Amer R; 2018; Jordan (44) | Cross-sectional; clinical setting | 220; People with T2DM | Depression | PHQ-9; | Using SEM:  1. **Direct** relationship between self-efficacy and self-management management behaviour  2. Depression was **indirectly** related to self-management management behaviour through self-efficacy 3. Social support was **indirectly** related to self-management management behaviour through depression |
|  |  |  | Illness Perception | BIPQ |  |
|  |  |  | Social support | ESSI |  |
|  |  |  | Coping | RSC |  |
|  |  |  | Self-management | SDSCA |  |
|  |  |  | Self-efficacy | DMSES |  |
| Devarajooh C; 2017; Malaysia (41) | Cross-sectional; clinical setting | 371; People with T2DM | Self-management | SDSCA | Using SEM: There was a significant direct positive effect from self-efficacy to diabetes self-management There were also significant direct adverse effects from depression and diabetes distress to diabetes self-efficacy.  Diabetes distress has a significant positive effect on depression. No significant direct association between both depression and diabetes distress with self-management; but depression and diabetes distress had a significant negative indirect effect on self-management via self-efficacy. |
|  |  |  | Self-efficacy | DMSES |  |
|  |  |  | Depression | PHQ-9 |  |
|  |  |  | Diabetes distress | DDS |  |
| Asuzu CC; 2017; USA (21) | Cross-sectional, clinical setting | 615; People with T2DM | Fatalism | DFS | Using SEM,  1. **Direct** relationship between diabetes distress and increased HbA1C  2. **Direct** relationship between diabetes distress and decreased self-management  3. **Indirect** association between increased depressive symptoms and decreased self-management through diabetes distress 4. **Indirect** association between increased fatalism and decreased self-management through diabetes distress |
|  |  |  | Depression | PHQ-9 |  |
|  |  |  | Self-management | SDSCA |  |
|  |  |  | Diabetes distress | DDS |  |
|  |  |  | Medication adherence | MMAS |  |
| Hernandez R; 2016; USA (35) | Cross-sectional, clinical setting | 250; T2DM of African American and Latino descent | Depression | PHQ-9 | Using Baron and Kenny method, *** 1. **A direct** relationship between depressive symptoms and diabetes self-management subcomponents of general diet, specific diet, physical activity, foot care, and smoking 2. The relationship between depression and foot care was mediated by self-efficacy among African Americans  3. Self-efficacy **did not mediate** the relationship between depression and any self-management areas among the Hispanic/Latino group |
|  |  |  | Self-management | SDSCA |  |
|  |  |  | Self-efficacy | DES-SF |  |
| Tovar E; 2015; USA (40) | cross-sectional, clinical setting | 201; People with T2DM | Depression | CES-D | Using Mediation analysis and Sobel test: 1. self-efficacy mediated the relationship between depression and adherence.  2. Social support (by spouse/significant other) does not mediate the association between depression and adherence  3. There is a significant indirect effect between depression and adherence via the social support provided by a doctor or healthcare team (p = .002)  4. Social support (by family and friends) mediates the relationship between depression and adherence |
|  |  |  | Self-efficacy | MDQ-SES |  |
|  |  |  | Social support | MDQ-SS |  |
|  |  |  | Self-management | SDSCA |  |
| Osborn CY; 2012; USA (36) | cross-sectional, clinical setting | 139; People with T2DM | Depression | PHQ-9 | Using the bootstrap method:  1. Direct relation between depression and medication nonadherence  2. Direct relation between depression and social support  3. Direct relation between social support and medication nonadherence  4. Partial indirect effect of depression on medication nonadherence through lack of social support |
|  |  |  | Social support | MOS |  |
|  |  |  | Medication adherence | MMAS |  |
| Sacco W; 2007; USA (51) | cross-sectional, clinical setting | 99; People with T2DM | Depression | PHQ-9 | Using mediation analysis (Sobel test):  1. **Diabetes symptoms** fully **mediate** the association between **BMI** and **depression**  2. **Self-efficacy** fully **mediated** the association between **BMI** and **depression**  **3. Self-efficacy** fully **mediated** the association between **adherence** and **depression**  **4.** The regression equation indicates that the effect of BMI on depression was nearly entirely accounted for by diabetes symptoms and self-efficacy, both of which independently contributed to depression  5. **Adherence did not mediate** the effect of depression on diabetes symptoms  6. **BMI did not mediate** the association between depression and diabetes symptoms  7. All study variables were significantly correlated with each other and with depression EXCEPT for adherence and diabetes symptoms |
|  |  |  | Self-management | SDSCA |  |
|  |  |  | Self-efficacy | MDQ-DSC |  |
|  |  |  | Diabetes Symptoms | DSC |  |
|  |  |  | BMI | Height and Weight |  |
| Harvey N; 2006; Canada (43) | cross-sectional, clinical setting; | 235; People with T2DM | Social support resources | CIRS | Using SEM:  1. Social-ecological support resources are directly related to diabetes self-management activity, accounting for 45% of the variance in diabetes self-management activity  2. Intra-individual variables (decreased emotional well-being, perceived personal and situational control) had indirect effects on diabetes self-management activity primarily through Social-ecological support resources |
|  |  |  | perceived locus of personal and situational health control | PCDS |  |
|  |  |  | self-management activity | SDSCA |  |
| Chao J; 2005; USA (47) | Cross-sectional, clinical setting | 445; People with T2DM on oral diabetes medication | Perceived benefit, barriers, susceptibility and severity | 18-item scale adapted from literatures | Using SEM,   - **Direct** relationship between depressive symptoms and perceived benefits of diabetes medication; perceived severity of diabetes and its complications; perceived general barriers; perceived side effect barriers, and self-efficacy - **Indirect** relationship between depressive symptoms and medication adherence **through** perceived general barriers, perceived side effect barriers, and self-efficacy |
|  |  |  | Self-efficacy | 1-item |  |
|  |  |  | Depression | PHQ-9 |  |
| McKellar J; 2004; USA (37) | Baseline cross-sectional data of an RCT clinical setting | 307; People with T2DM using hypo- glycemic medication | Depression | CES-D | Using SEM,  1. Depressive symptoms **have little direct** impact on **diabetes-related symptoms** above and beyond their impact on patients' **self-management behaviours** |
|  |  |  | Self-management adherence | MMAS |  |
|  |  |  | Diabetes Symptom Burden | Participants' reports |  |
| **BIPQ** – Brief Illness Perception Questionnaire; **PHQ-9** – Patient Health Questionnaire; **ESSI** – Enrich Social Support Instrument; **RSC** – Diabetes Fatalism Scale – Religious and Spiritual Coping; **SDSCA** – Summary of Diabetes Self‐Care Activities; **PAID** – Problem Areas in Diabetes; **DMSES** – Diabetes Management Self‐Efficacy Scale; **DDS** – Diabetes Distress Scale; **DFS** - Diabetes Fatalism Scale; **DES-SF** - Diabetes Empowerment Scale-Short Form; **MOS** – Medical Outcomes Study - Social Support Survey; **MMAS** – Morisky Medication Adherence Scale; **PCDS** – Perceived Competence for Diabetes Scale; **CES-D** - Center for Epidemiological Studies-Depression Scale; **MDQ-SES** -Multidimensional Diabetes Questionnaire Self-Efficacy subscale; **DSC** – Diabetes Symptom Checklist; **CIRS** – Chronic Illness Resources Survey; **DWBQ** – Diabetes Well-being Questionnaire; **IPQ-R** -The revised Illness Perception Questionnaire; **DUFSSQ** – Duke-UNC Functional Social Support Questionnaire; **ADDQoL** – Audit of Diabetes Dependent Quality of Life; **PMS** – Pearlin Mastery Scale; **OPQoL** – Older People's Quality of Life Questionnaire; **MSPSS** – Multidimensional Scale of Perceived Social Support; **DASS-21** – Depression Anxiety Stress Scale | | | | | |

* Hayes' approach to mediation analyses uses bias-corrected bootstrap confidence intervals (bootstrapping = 10,000) to estimate and interpret the effect size of the direct and indirect effects of the independent variable on the dependent variable

*** The Baron and Kenny method is a statistical analysis strategy for testing mediation.
